# Cardiac resynchronization therapy and its effects in patients with type 2 DIAbetes mellitus OPTimized in automatic vs. echo guided approach. Data from the DIA-OPTA investigators

**DOI:** 10.1186/s12933-020-01180-8

**Published:** 2020-11-28

**Authors:** Celestino Sardu, Pasquale Paolisso, Valentino Ducceschi, Matteo Santamaria, Cosimo Sacra, Massimo Massetti, Antonio Ruocco, Raffaele Marfella

**Affiliations:** 1 Department of Advanced Medical and Surgical Sciences, University of Campania “Luigi Vanvitelli”, Piazza Miraglia 2, 80131 Naples, Italy; 2 Unit of Cardiology, Department of Experimental, Diagnostic and Specialty Medicine-DIMES, University of Bologna, Bologna, Italy; 3 Unit of Cardiovascular Diseases and Arrhythmias, “Vecchio Pellegrini” Hospital, Naples, Italy; 4 Unit of Cardiovascular Diseases and Arrhythmias, “Gemelli Molise”, Campobasso, Italy; 5 Department of Cardiac Surgery and Cardiovascular Diseases, “Catholic University of Sacred Heart”, Rome, Italy; 6 Unit of Cardiovascular Diseases and Arrhythmias, “Antonio Cardarelli” Hospital, Naples, Italy

**Keywords:** Type 2 diabetes mellitus, Cardiac resynchronization therapy, Automatic CRTd optimization

## Abstract

**Objectives:**

To evaluate the effects of cardiac resynchronization therapy (CRTd) in patients with type 2 diabetes mellitus (T2DM) optimized via automatic vs. echocardiography-guided approach.

**Background:**

The suboptimal atrio-ventricular (AV) and inter-ventricular (VV) delays optimization reduces CRTd response. Therefore, we hypothesized that automatic CRTd optimization might improve clinical outcomes in T2DM patients.

**Methods:**

We designed a prospective, multicenter study to recruit, from October 2016 to June 2019, 191 consecutive failing heart patients with T2DM, and candidate to receive a CRTd. Study outcomes were CRTd responders rate, hospitalizations for heart failure (HF) worsening, cardiac deaths and all cause of deaths in T2DM patients treated with CRTd and randomly optimized via automatic (n 93) vs. echocardiography-guided (n 98) approach at 12 months of follow-up.

**Results:**

We had a significant difference in the rate of CRTd responders (68 (73.1%) vs. 58 (59.2%), p 0.038), and hospitalizations for HF worsening (12 (16.1%) vs. 22 (22.4%), p 0.030) in automatic vs. echocardiography-guided group of patients. At multivariate Cox regression analysis, the automatic guided approach (3.636 [1.271–10.399], CI 95%, p 0.016) and baseline highest values of atrium pressure (automatic SonR values, 2.863 [1.537–6.231], CI 95%, p 0.006) predicted rate of CRTd responders. In automatic group, we had significant difference in SonR values comparing the rate of CRTd responders vs. non responders (1.24 ± 0.72 g vs. 0.58 ± 0.46 g (follow-up), p 0.001), the rate of hospitalizations for HF worsening events (0.48 ± 0.29 g vs. 1.18 ± 0.43 g, p 0.001), and the rate of cardiac deaths ( 1.13 ± 0.72 g vs. 0.65 ± 0.69 g, p 0.047).

**Conclusions:**

Automatic optimization increased CRTd responders rate, and reduced hospitalizations for HF worsening. Intriguingly, automatic CRTd and highest baseline values of SonR could be predictive of CRTd responders. Notably, there was a significant difference in SonR values for CRTd responders vs. non responders, and about hospitalizations for HF worsening and cardiac deaths.

*Clinical trial* ClinicalTrials.gov Identifier NCT04547244.

## Background

Type 2 diabetes mellitus (T2DM) is a risk factor, that negatively impacts on clinical prognosis for patients with heart failure (HF), and in those receiving a Cardiac resynchronization therapy with defibrillator (CRTd), [1]. On other hand, the CRTd could ameliorate clinical outcomes, because it has a positive impact on both morbidity and mortality in treated patients [2]. Notably, the T2DM accounts about the 38% of patients treated with a CRTd [1], and the patients which do not respond to CRTd are defined “CRTd non responders”, and are those with worse prognosis [3]. In this setting, the T2DM is a leading cause of multiple and complex alterations of molecular, metabolic, electrical, and mechanical cardiac functions, which cause arrhythmias and worsening of cardiac pump [1]. Consequently, the worsening of cardiac pump is a relevant cause of hospitalizations and deaths in CRTd patients [1–5]. Thus, in last decades a great effort has been invested to develop new therapeutic approaches to improve the cardiac pump efficiency, the number of CRTd responders and the clinical outcomes in CRTd patients with T2DM. In this setting, the use of multipolar left-ventricular (LV) pacing leads, and the optimization of CRTd device programming mode has been seen as an important advancement in T2DM patients with CRTd [6, 7, 8]. On other hand, also T2DM patients receiving a multipolar CRTd could experience a worse prognosis [6]. This could be caused by the reduction of cardiac pump, which is more evidenced in patients with the loss of atrio-ventricular (AV) and inter-ventricular (IV) synchrony [9]. Therefore, the optimization of AV and IV intervals could be a therapeutic target, to ameliorate the CRTd effectiveness, and to increase the rate of CRTd responders [8, 9]. By the way, the echocardiography could be used to ameliorate the optimization of AV/IV intervals [9]. On other hand, echocardiography showed contrasting results in clinical studies, and low application in clinical practice [9]. Therefore, new techniques, as the intracardiac electrogram (IEGM) guided approach, have been proposed for the optimization of AV/IV intervals in CRTd patients [9]. Indeed, the IEGM-guided approach is faster, simpler, and it is a reliable alternative to the echo-guided approach for CRTd optimization [9]. On the other hand, the IEGM-guided approach showed contrasting results in the optimization of the CRTd [9]. Furthermore, authors showed its inferiority as compared to echocardiography-guided approach about the hemodynamic outcome [9]. Therefore, recently authors have introduced a new optimization technique, that is not IEGM-guided [8]. This new optimization technique is correlated with dP/dt max of LV, and with the hemodynamic function of the heart [8]. To date, this non IEGM-guided technique evaluates the peak of endocardial acceleration during isovolumetric contraction of the left ventricle, and its amplitude, that is recorded as SonR signal [8, 10]. Therefore, the values of SonR are related to the contractile function of the heart [8], and the automatic vs. echo-guided approach could lead to an increase of response to the CRTd [10]. However, our study hypothesis was that automatic vs. echo-guided approach might result in best optimization of AV/IV delays in T2DM patients treated by multipolar CRTd. In addition, changes in SonR signals could be seen in diabetic patients with CRTd who experienced the main clinical outcomes. Thus, automatic vs. echo-guided CRTd optimization could result in reduction of CRTd responders, of hospitalizations for HF worsening and deaths (cardiac deaths and all causes of death) in HF patients with T2DM treated with multipolar CRTd. Therefore, in the present study we assessed the rate of CRTd responders, the hospitalizations for HF worsening and the deaths (cardiac deaths and all causes of death) in HF patients with T2DM treated with multipolar CRTd, and randomly assigned to the automatic vs. echo-guided group of CRTd optimization at 12 months of follow-up. Finally, we assessed the SonR values at baseline and at follow-up of 12 months for CRTd responders, for patients with hospitalizations for HF worsening, and for deaths events.

## Methods

### Study design

**Fig. 1:**
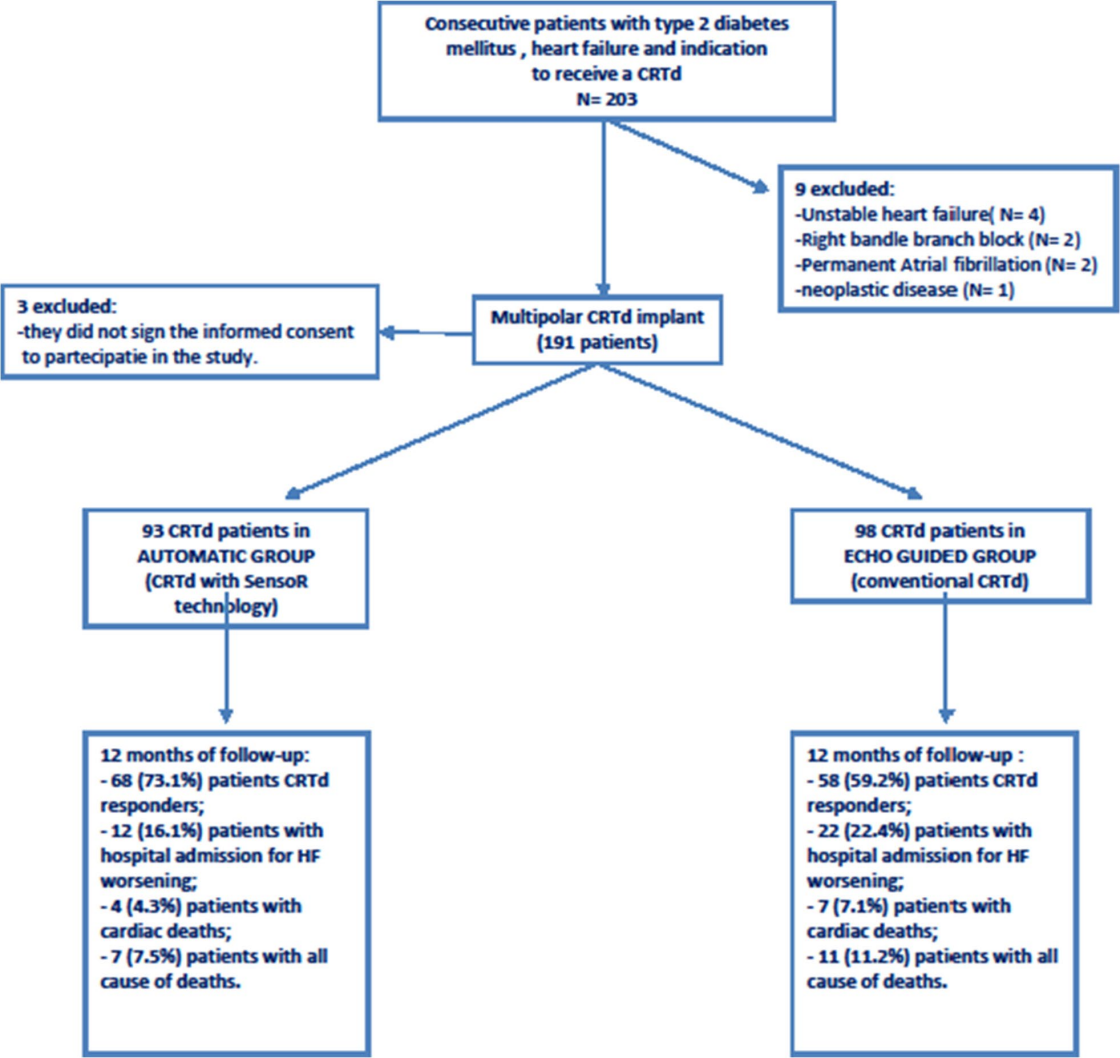
Representation of study flow chart. CRTd: cardiac resynchronization therapy with a defibrillator; HF: heart failure

Between 11th January 2010 and 20th January 2019, we screened a population of 203 consecutive patients with T2DM, chronic HF and indication to receive a CRTd in an observational multicenter, randomized study (DIA-OPTA investigators) Fig. 1. The diagnosis of T2DM was made according to American Diabetes Association criteria [11]. To establish T2DM patients treatment, the screened patients answered a specific questionnaire about medicines used for diabetes treatment, with the date of the beginning and end of treatment, route of administration, and duration of use [11]. The diagnosis of HF was made as indicated by international guidelines on HF disease management [12]. Moreover, only patients with T2DM and HF were enrolled in the study, according to inclusion/exclusion criteria. Figure 1. The study population respected the following inclusion/exclusion criteria:

*Inclusion criteria*: at least 18 years of age, T2DM diagnosis, with clinical history of stable chronic heart failure, New York Heart Association (NYHA) functional class II or III, sinus rhythm, left bundle branch block, severe left ventricle ejection fraction reduction (LVEF < 35%), stable sinus rhythm, and candidates to receive a CRT-d treatment [12].

*Exclusion criteria:* age < 18 or > 75 years, ejection fraction > 35%, previous implant of implantable cardioverter defibrillator (ICD), CRT-d and/or pacemaker, absence of informed patient consent, and any condition that would make survival for 1 year unlikely.

### Study population and intervention

The 191 enrolled patients with T2DM and HF respected the clinical indication for implantation of a de-novo multipolar CRTd, according to current international guidelines [12].

Thus, we randomly treated the patients with T2DM via conventional CRTd implant (n 98) vs. SensoR-CRTd (n 93), using a computer generating a code program. The patients with conventional CRTd implant were optimized using echo-guided approach, and defined as “Echo group”. The patients with automatic sensor guided CRTd were defined as “Automatic group”, and they were not optimized by echo-guided approach Fig. 1. However, in a time of 14 days after a successful CRTd implant, patients were randomized (2:1, respectively) to weekly automatic AV and IV delay optimization with SonR in Automatic group vs. echo-guided optimization in Echo group. The full description of CRTd implant (Automatic vs. Echo group) is provided in Additional file 1.

At baseline and for all follow-up duration (6 and 12 months) the patients underwent full echocardiographic evaluation, and a global clinical status (NYHA) assessment, and CRTd device interrogation [13]. Before CRTd intervention and during follow-up, we determined the baseline laboratory studies by peripheral blood and enzymatic assays after an overnight fast (values of plasma glucose, glycated hemoglobin 1Ac type (HbA1c), B type natriuretic peptide (BNP) and serum lipids). In addition, at baseline, and during follow up we measured inflammatory markers as circulating serum levels of pro-inflammatory cytokines (tumor necrosis factor-α, TNF α, interleukin-6, IL6), systemic inflammatory markers (C reactive protein, CRP), and leucocytes and neutrophils count as previously reported [13].

Thereafter, for each enrolled patient during clinical, instrumental assessment, and device telemetric control (at implant, 10 days, 6, and 12 months after discharge) and by visualization of hospital discharge schedules, we reported the effects of CRT-d in terms of clinical outcomes, CRT responders rate, and clinical events as hospitalizations for HF worsening, and deaths. The full description is reported in Additional file 1.

### Echocardiographic evaluation

Two experienced physicians in echocardiography (Co. S, M.M), performed at baseline, and at 6th and 12th month of follow up, a trans-thoracic two-dimensional echocardiogram with M-mode, conventional Doppler, and pulsed-wave tissue Doppler imaging (TDI) measurements in each patient using a Philips iE33 echocardiograph (Eindhoven, The Netherlands). The images of echocardiography were acquired in the parasternal long and short axis views. However, we calculated LV end-diastolic diameter (LVEDD), end-diastolic volume (LVEDV), end-systolic diameter (LVESD), end-systolic volume (LVESV), and then we determined LV ejection fraction (LVEF) with the Simpson biplane method [14]. In addition, the amount of mitral regurgitation was calculated as the area of the color-flow Doppler regurgitant jet divided by the area of the left atrium in systole, and described as low ( +), moderate (+ +), moderate-severe (+ + +), and severe (+ +  + +), [14, 15]. To have final calculation measures the physicians performed systematically averaged measurements in five consecutive samples. The physicians involved in echocardiographic baseline and follow-up evaluation, performed and analyzed each exam in independent way, and blinded to the study protocol. In a time of 14 days after a successful CRTd implant, patients were randomized (2:1, respectively) to the automatic atrio-ventricular (AV) and inter-ventricular (IV) delay optimization with SonR (Automatic group) vs. Echo-guided optimization (Echo group), using a mandatory standardized protocol [16, 17]. At baseline and at follow-up, we performed the echocardiography measurements in CRTd patients at rest, and in supine position [12]. Finally, all measurements were reviewed by two observers blinded to measures performed previously by other observers. In addition, the observers were blinded to study protocol and to the group of study that the patient had been selected for.

### Evaluation of SonR signals and automatic optimization of AV/IV intervals

After CRTd implant the patients in Automatic and Echo group were evaluated by device interrogation at follow-up, as previously described by authors [10]. Specifically, for patients in the automatic group, during CRTd interrogation, we evaluated the modifications (baseline vs. follow-up values) of signals recorded by SonR sensor, that is allocated in right atrium lead [10]. Intriguingly, the values of SonR signals are recordings of endocardial acceleration signals of the heart, that correspond to the mechanical vibrations of myocardium during cardiac contraction [8, 10]. Thus, during the isovolumetric contraction phase of the cardiac cycle we registered the highest amplitude of the SonR signal, that correspond to the cardiac contractility [10]. However, there is a correlation between the amplitude of the recorded SonR signal and LV dP/dtmax, and so it could be seen as index of the contractile function of the heart [10]. Indeed, the amplitude of SonR signal corresponds to the first heart sound, and it is a surrogate index of systolic function of heart [10]. Furthermore, from registration and evaluation of SonR signals, the CRT-d automatically adjusts the AV/IV delays, on a weekly basis, at rest and during exercise in the automatic group [10]. Finally, a concordance has been showed between echocardiographic methods and SonR device based method used for AV/IV delays optimization [10].

### Echocardiographic optimization of the atrioventricular and interventricular intervals

In the echo-guided group, we optimized the AV and IV intervals via echocardiography during continuous ECG monitoring in each patient, and guided by a careful analysis of the 12 -lead ECG [16]. For AV interval optimization we followed the recommendations of the American Society of Echocardiography, via the simplified pulsed Doppler mitral inflow technique [16]. Thus, the AV delay optimization was performed by the evaluation of trans mitral flow using the pulsed wave Doppler [16, 17]. Moreover, we then optimized the LV diastolic filling so that mitral valve closure coincided with the end of the Doppler A wave during ventricular systole [17]. However, it was integrated with the optimized AV delay by the aortic velocity–time integral (VTI) method, by assessing the VTI of flow across the aortic valve [17]. Indeed, VTI measures are directly proportional to LV stroke volume [16, 17]. Thus, we programmed the VV interval as AV delay optimization by using the aortic VTI method [17]. However, after the determination of the optimal AV delay programming, we performed the VV interval optimization to decrease LV dyssynchrony, by providing a more simultaneous LV activation and reducing the mitral regurgitation in some patients [17].

### Study endpoints

Primary endpoints were the rate of CRTd responders comparing patients in Automatic vs. Echo group. Secondary study endpoints were the hospitalizations for HF worsening, cardiac deaths and all cause of deaths events comparing patients in Automatic vs. Echo group. In addition, in Automatic group of patients we evaluated the amplitude of SonR signals at baseline, and their variations at follow-up for the CRTd responders vs. non responders, and for the events of hospitalization for HF worsening, cardiac deaths and all cause of deaths.

### Definition of CRTd responders

CRT responders were defined, according to authors, by evidence of clinical and echocardiographic diagnostic criteria [12]. Thus, clinically the CRTd responders showed the improvement in NYHA functional class (at least one class) and the increase of the 6 min walk distance > 10%, [12]. At echocardiography, the CRTd responders showed a reduction LVESD > 15%, and an improvement in LVEF > 10%, [12]. In addition to clinical and instrumental evaluation, authors identified CRTd responders patients also by chest X-rays, to assess reduction in cardiac size and pulmonary congestion [12].

The primary and secondary study endpoints were evaluated at follow-up of 12 months during visits and controls, and by hospital discharge schedules. The detailed description of secondary study endpoint diagnostic criteria, and of study endpoints data collection and analysis was reported in Additional file 1.

### Ethical Committee and Clinical trial registration

Authors conducted the study in accordance with the Declaration of Helsinki. The Ethics Committees of all participating institutions approved the protocol. All patients were informed about the study nature, and gave their written informed, and signed consent to participate in the study. The study was registered in ClinicalTrials.gov, clinical trial number NCT04547244. The authors and investigators of DIA-OPTA study accepted full responsibility for the accuracy and completeness of the data and all analyses, and for the fidelity of this report of the trial protocol.

### Statistical analysis

The collected data were analyzed by a qualified statistician. The T2DM patients with CRTd were divided into automatic vs. echo group of patients (conventional group or controls), and during follow up visits, and controls in CRT-d responders vs. CRT-d non-responders. Moreover, we supposed that the number of patients with alterations in primary and secondary endpoints was significantly different between the two groups of patients. Safety analyzes were performed on data from all enrolled patients. Thus, we expressed the continuous variables as means and standard deviations, that were tested by two-tailed Student t test for paired or unpaired data, as appropriate, or by one-way analysis of variance (ANOVA) for more than two independent groups of data. The categorical variables were compared by Chi square or Fisher exact test where appropriate. We performed survival analysis by the Kaplan Meier method, and we evaluated the predictors of the study endpoints by Cox regression models in patients with automatic as compared with echo-guided CRTd. However, we conducted an univariate analysis to examine the association between single principal clinic, echocardiographic, electrocardiographic characteristics, etc. and automatic CRTd effects, and 12 months study outcomes (CRTd responders rate, hospitalizations for HF worsening, all cause of deaths and cardiac deaths). However, Cox models were adjusted for; age, Body mass index, cholesterol, dyslipidemia, beta-blockers, ace-inhibitors, calcium inhibitors, etc. Therefore, only variables presenting a p value ≤ 0.25 at the univariate analysis were included in the model. We used a stepwise method with backward elimination. and we calculated odds ratios (OR) with 95% confidence intervals. The model was evaluated with Hosmer and Lemeshow test. A 2-sided p < 0.05 was considered statistically significant. The statistical analysis was performed using the SPSS software package for Windows 17.0 (SPSS Inc., Chicago Illinois).

## Results

In the present study we analyzed 191 T2DM patients with multipolar CRTd, divided in Automatic group (n 93), vs. Echo group (conventional CRTd implant, n 98) Fig. 1. Characteristics of study population at baseline were reported in Table 1.

**Table 1:** Clinical characteristics of study population at baseline in overall, and automatic vs. echo-guided patients

Parameters	Overall population (n 191)	Automatic (n 93)	Echo guided (n 98)	P value
Age	71 ± 6	71 ± 7	72 ± 6	0.426
Male (%)	134 (70.2)	63 (67.7)	71 (72.4)	0.431
Smokers (%)	97 (50.8)	46 (49.5)	51 (52)	0.407
Hypertension (%)	136 (71.2)	65 (69.9)	71 (72.5)	0.282
Dislipidemia (%)	71 (37.2)	35 (37.6)	36 (36.7)	0.462
Plasma glucose (mg/dl)	186.7 ± 22.1	185.5 ± 22.3	188.9 ± 22.0	0.367
HbA1c (mmol/mol)	57.9 ± 16.3	57.8 ± 16.2	58.1 ± 16.4	0.263
BMI > 30 kg/m^2^(%)	15 (7.8)	8 (8.6)	7 (7.1)	0.791
COPD (%)	35 (18.3)	17 (18.3)	18 (18.4)	0.538
Renal disease (%)	35 (18.3)	16 (17.2)	19 (19.4)	0.105
Ischemic heartfailure (%)	131 (68.6%)	65 (69.9)	66 (67.4)	0.302
II NYHA class (%)	33 (25.2)	16 (24.6)	17 (25.8)	0.280
III NYHA class (%)	98 (74.8)	49 (75.4)	49 (74.2)	0.211
QRS duration (ms)	137.4 ± 9.2	137.5 ± 9.0	137.9 ± 9.4	0.930
6MWT	243.47 ± 41.83	241.18 ± 44.94	246.75 ± 40.74	0.371
SonR values (g)	/	0.24 ± 0.08	/	/
Echocardiographic parameters				
LVEF (%)	27 ± 8	27 ± 5	28 ± 5	0.285
LVEDd (mm)	65 ± 8	66 ± 7	64 ± 9	0.101
LVESd (mm)	43 ± 8	41 ± 6	44 ± 9	0.291
LVEDv (ml)	205 ± 20	206 ± 18	203 ± 22	0.993
LVESv (ml)	146 ± 17	148 ± 15	145 ± 18	0.818
Mitral insufficiency				
+ (%)	96 (50.3)	45 (48.4)	51 (52.0)	0.359
+ + (%)	78 (40.8)	38 (40.9)	40 (40.8)	0.556
+ + + (%)	17 (8.9)	10 (10.7)	7 (7.2)	0.451
Medications at baseline				
Amiodarone (%)	40 (20.9)	19 (20.4)	21 (21.4)	0.569
ACE inhibitors (%)	86 (45)	42 (45.2)	44 (44.9)	0.543
ARS blockers (%)	61 (31.9)	31 (33.3)	30 (30.6)	0.464
Sacubitril/valsartan (%)	47 (24.6)	23 (24.7)	24 (24.5)	0.551
Beta blockers:				
Carvedilol (%)	74 (38.7)	36 (38.7)	38 (38.8)	0.555
Bisoprolol (%)	62 (32.5)	32 (34.4)	30 (30.6)	0.539
Aspirin (%)	76 (39.8)	36 (38.7)	40 (40.8)	0.558
Tiklopidine(%)	5 (2.6)	2 (2.1)	3 (3.1)	0.525
Warfarin (%)	57 (29.8)	27 (29)	30 (30.6)	0.468
NOAC (%)	45 (23.6)	20 (21.5)	25 (25.5)	0.316
Calcium antagonist (%)	12 (6.3)	5 (5.4)	7 (7.1)	0.501
Ivabradine(%)	40 (20.9)	21 (22.6)	19 (19.4)	0.599
Digoxin (%)	57 (29.8)	27 (29)	30 (30.6)	0.468
Loop diuretics (%)	168 (88)	79 (84.9)	89 (90.8)	0.268
Aldosterone Blockers (%)	117 (61.3)	55 (59.1)	62 (63.3)	0.656
Statins (%)	142 (74.3)	69 (74.2)	73 (74.5)	0.461
Anti diabetic drugs, n (%)				
Insulin (%)	40 (20.9)	18 (19.3)	22 (22.4)	0.722
Metformin (%)	109 (57.1)	49 (52.7)	60 (61.2)	0.246
Sulfonylureas (%)	34 (17.8)	16 (17.2)	18 (18.4)	0.852
Thiazolidinediones (%)	22 (11.5)	10 (10.7)	12 (12.2)	0.823
GLP-1 agonist (%)	28 (14.7)	13 (14)	15 (15.3)	0.840
DPP-4 inhibitors (%):	40 (20.9)	18 (19.3)	22 (22.5)	0.722
Biomarkers				
Lymphocytes	7.95 ± 2.29	7.99 ± 2.23	7.83 ± 2.35	0.271
Neutrophiles	5.38 ± 1.92	5.40 ± 1.95	5.37 ± 1.90	0.421
BNP (pg/ml)	327.38 ± 18.61	321.04 ± 18.72	332.74 ± 19.55	0.667
CRP (mg/L)	9.84 ± 0.94	10.36 ± 1.03	9.36 ± 0.97	0.466
IL6 (pg/ml)	6.42 ± 0.05	6.38 ± 0.04	6.47 ± 0.06	0.272
TNFα (pg/ml)	6.31 ± 0.03	6.34 ± 0.03	6.29 ± 0.02	0.269

ACE, Angiotensin Converting Enzyme; ARS, Angiotensin Receptors; BMI, body mass index; BNP, B type natriuretic peptide; COPD, chronic obstructive pulmonary disease; CRP, C reactive protein; DPP-4, dipeptidyl peptidase-4; HbA1c, glycated hemoglobin 1Ac type; GLP-1, glucagone like peptide-1; IL-6, interleukine 6; LVEDd, left ventricle end diastolic diameter; LVEDv, left ventricle end diastolic volume; LVEF, left ventricle ejection fraction; LVESd, left ventricle end systolic diameter; LVESv, left ventricle end systolic volume; NYHA II, III, New York Heart Association II and III class; NOAC, new oral anti coagulation; SonR, values of SonR signals; TNFα, tumor necrosis factor alpha; 6MWT, 6 min walking test.* is for statistical significant (p < 0.05)

*At 12th month of follow up,* patients in automatic vs. echo group showed a significant reduction of NYHA class, BNP values (148.41 ± 16.40 vs. 197.26 ± 19.12 pg/ml, p 0.001), and inflammatory markers values, with higher values of 6MWT (319.37 ± 26.92 vs. 227.92 ± 28.19), significant reduction of left ventricle systolic diameters/volumes and mitral valve insufficiency (p < 0.05), and significant improvement of LVEF (36 ± 6 vs. 27 ± 5, p 0.001) Table 2.

**Table 2:** Clinical characteristics of study population at 12th month of follow-up in overall, and automatic vs. echo-guided patients

Parameters	Automatic (n 93)	Echo guided (n 98)	P value
12 months follow up			
BMI > 30 kg/m^2^(%)	7 (7.5)	6 (6.1)	0.622
Plasma glucose (mg/dl)	173.5 ± 21.7	171.2 ± 20.9	0.171
HbA1c (mmol/mol)	52.9 ± 12.1	52.3 ± 12.0	0.122
I NYHA class	6 (6.4)	2 (2.0)	0.016*
II NYHA class	45 (48.4)	21 (21.4)	0.010*
III NYHA class	38 (40.9)	66 (67.3)	0.001*
IV NYHA class	4 (4.3)	10 (10.2)	0.021*
QRS duration	121.6 ± 9.6	122.9 ± 9.1	0.251
6MWT	319.37 ± 26.92	227.92 ± 28.19	0.005*
SonR values (g)	1.09 ± 0.07	/	/
Echocardiographic parameters			
LVEF (%)	36 ± 6	27 ± 5	0.001*
LVEDd (mm)	63 ± 5	65 ± 8	0.051
LVESd (mm)	35 ± 4	38 ± 5	0.001*
LVEDv (ml)	165 ± 24	178 ± 41	0.054
LVESv (ml)	109 ± 12	126 ± 18	0.001*
Mitral insufficiency			
+ (%)	50 (53.8)	31 (31.6)	0.040*
+ + (%)	38 (40.9)	57 (58.2)	0.004*
+ + + (%)	5 (5.4)	10 (10.2)	0.285
Biomarkers			
Lymphocytes	7.12 ± 1.27	8.48 ± 1.18	0.001*
Neutrophiles	4.87 ± 1.85	5.69 ± 2.31	0.001*
BNP (pg/ml)	148.41 ± 16.40	197.26 ± 19.12	0.001*
CRP (mg/L)	7.24 ± 0.56	8.69 ± 0.83	0.036*
IL6 (pg/ml)	5.55 ± 0.03	6.31 ± 0.03	0.011*
TNFα (pg/ml)	5.35 ± 0.02	6.31 ± 0.02	0.005*
Study outcomes			
CRTd responders (%)	68 (73.1)	58 (59.2)	0.038*
Hospital admission for HF worsening (%)	12 (16.1)	22 (22.4)	0.030*
Cardiac deaths (%)	4 (4.3)	7 (7.1)	0.538
All cause of deaths (%)	7 (7.5)	11 (11.2)	0.461

BMI, body mass index; BNP, B type natriuretic peptide; CRP, C reactive protein; HbA1c, glycated hemoglobin 1Ac type; IL-6, interleukine 6; LVEDd, left ventricle end diastolic diameter; LVEDv, left ventricle end diastolic volume; LVEF, left ventricle ejection fraction; LVESd, left ventricle end systolic diameter; LVESv, left ventricle end systolic volume; NYHA II, III, New York Heart Association II and III class; SonR, values of SonR signals; TNFα, tumor necrosis factor alpha; 6MWT, 6 min walking test.** is for statistical significant (p < 0.05)

*As primary study endpoints,* comparing patients in automatic vs. echo group, we had a significant higher rate of CRTd responders (68 (73.1%) vs. 58 (59.2%), p value 0.038) at 12 months of follow-up Table 2.

*As secondary study endpoints*, comparing patients in automatic vs. echo group, we had a significant lower rate of hospitalizations for HF worsening (12 (16.1%) vs. 22 (22.4%), p value 0.030) at 12 months of follow-up Table 2.

**Fig. 2:**
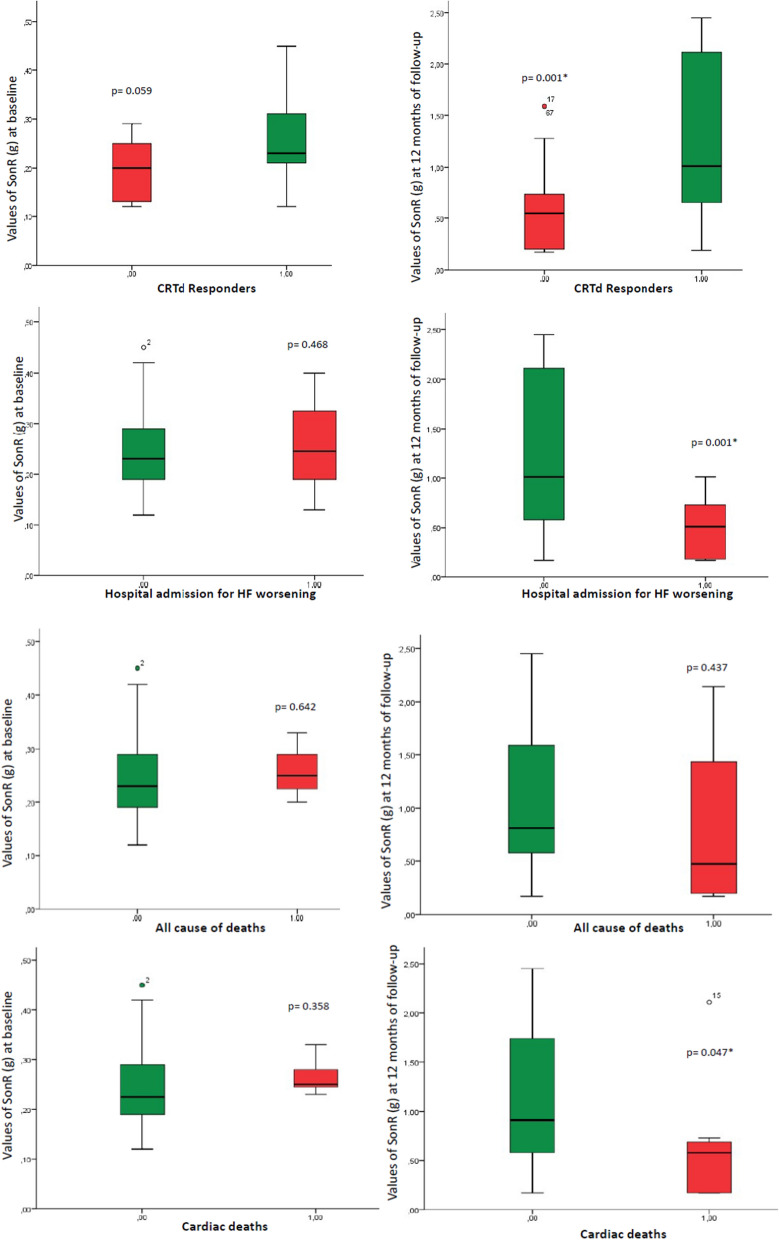
**a** In upper part the SonR values (g) at baseline (left part) and at follow-up end in CRTd responders (green color) vs. CRTd non responders (red color) with the corresponding p value. In lower part the SonR values in g at baseline (left part) and at follow-up end in patients with hospital admission for heart failure (HF) worsening (red color) vs. patients without hospital admission for heart failure (HF) worsening (green color) with the corresponding p value. * is for statistical significant (p < 0.05). **b** In upper part the SonR values in g at baseline (left part) and at follow-up end for patients with all cause of deaths (red color) vs. survived patients (green color) with the corresponding p value. In lower part the SonR values in g at baseline (left part) and at follow-up end in patients with cardiac deaths (red color) vs. survived patients (green color) with the corresponding p value. * is for statistical significant (p < 0.05)

Intriguingly, at baseline in the automatic group of CRTd patients we did not find a significant difference in SonR values comparing CRTd responders vs. non responders (0.27 ± 0.07 g vs. 0.195 ± 0.05 g, p 0.055), the patients with vs. those without hospital admissions for HF worsening (0.25 ± 0.08 g vs. 0.24 ± 0.08 g, p 0.468), the patients with vs. those without all cause of deaths (0.26 ± 0.05 g vs. 0.24 ± 0.08 g, p 0.642) and the patients with vs. those without cardiac deaths (0.27 ± 0.04 g vs. 0.24 ± 0.08 g, p 0.358). Figure 2. At follow-up end, this trend was confirmed only for all cause of deaths (0.81 ± 0.19 g vs. 1.10 ± 0.08 g, p 0.437), while there was a statistical significant difference about SonR values comparing CRTd responders vs. non responders (1.24 ± 0.72 g vs. 0.58 ± 0.46 g (follow-up), p 0.001), hospital admissions for HF worsening events (0.48 ± 0.29 g vs. 1.18 ± 0.43 g, p 0.001), and cardiac deaths ( 1.13 ± 0.72 g vs. 0.65 ± 0.69 g, p 0.047) Fig. 2.

At multivariate Cox regression analysis, automatic CRTd (HR 3.636, [1.271–10.399] CI 95%, p 0.016), and baseline SonR values (HR 2.863, [1.537–6.231] CI 95%, p 0.006) were predictors of CRTD responders rate Tables 3.

**Table 3:** Univariate and Multivariate Cox regression analysis for CRTd responders (a), hospitalization for HF worsening (b), cardiac deaths (c) and all cause of deaths (d)

	Univariate analysis HR (95% CI)	p value	Multivariate analysis HR (95% CI)	p value
A. Multivariate Cox regression analysis for parameters associated with CRT responders				
Age	0.102 [0.11–0.968]	0.048	0.713 [0.007–1.773]	0.276
Automatic	0.795 [0.567–1.115]	0.184	*3.636 [1.271–10.399]*	0.016*
Beta blockers	1.176 [0.806–1.716]	0.401	1.156 [0.745–1.793]	0.517
BNP	1.001 [0.989–1.101]	0.868	1.001 [0.889–1.007]	0.816
COPD	1.446 [0.948–2.204]	0.087	1.527 [0.935–2.495]	0.091
CRP	1.101 [0.992–1.280]	0.274	1.017 [0.995–1.041]	0.136
HbA1c	1.118 [0.851–1.315]	0.643	1.181 [0.922–1.472]	0.123
Hypertension	0.898 [0.619–1.302]	0.569	0.895 [0.561–1.430]	0.644
LVEF	1.006 [0.970–1.044]	0.736	1.036 [0.993–1.081]	0.102
NYHA 3	1.176 [0.840–1.647]	0.345	1.829 [0.923–3.626]	0.084
Obesity	1.497 [1.290–1.852]	0.011	1.330 [0.829–1.843]	0.082
QRS duration	0.989 [0.971–1.008]	0.255	0.991 [0.971–1.011]	0.379
SonR	10.2 [5.227–19.952]	0.002	*2.863 [1.537–6.231]*	0.006*
6MWT	1.001 [0.993–1.007]	0.926	1.010 [0.993–1.007]	0.994
B. Multivariate Cox regression analysis for parameters associated with hospitalizations for heart failure				
Age	1.012 [0.873–1.322]	0.958	0.997 [0.671–1.201]	0.240
Automatic	0.795 [0.567–1.115]	0.184	1.166 [0.118–1.504]	0.895
Beta blockers	1.301 [0.587–2.885]	0.517	0.844 [0.336–2.122]	0.718
BNP	1.002 [1.001–1.301]	0.011	1.002 [1.001–1.040]	0.125
COPD	0.561 [0.276–1.141]	0.111	2.364 [0.907–6.158]	0.078
CRP	1.011 [0.978–1.046]	0.507	1.032 [0.982–1.084]	0.219
HbA1c	1.142 [0.816–1.913]	0.143	0.915 [0.589–1.541]	0.762
Hypertension	1.991 [1.003–3.952]	0.049	2.503 [0.809–7.745]	0.111
LVEF	1.029 [0.956–1.108]	0.443	1.061 [0.960–1.172]	0.245
NYHA 3	0.531 [0.258–1.096]	0.087	0.962 [0.289–3.202]	0.950
Obesity	0.905 [0.276–2.965]	0.869	1.093 [0.220–5.429]	0.913
QRS duration	0.964 [0.927–1.020]	0.066	0.960 [0.919–1.003]	0.069
SonR	0.074 [0.004–1.292]	0.074	0.679 [0.118–1.154]	0.932
6MWT	0.999 [0.985–1.013]	0.891	0.995 [0.980–1.011]	0.552
C. Multivariate Cox regression analysis for parameters associated with cardiac deaths				
Age	0.953 [0.795–1.541]	0.543	1.362 [0.632–1.872]	0.361
Automatic	1.670 [0.489–5.705]	0.413	0.111 [0.001–2.583]	0.427
Beta blockers	2.099 [0.525–3.024]	0.176	1.095 [0.048–1.435]	0.945
BNP	1.001 [0.998–1.004]	0.681	0.999 [0.996–1.003]	0.679
COPD	1.119 [0.031–1.447]	0.072	2.138 [0.942–4.002]	0.401
CRP	0.913 [0.807–1.034]	0.151	0.903 [0.734–1.112]	0.338
HbA1c	1.601 [0.925–2.563]	0.142	3.224 [0.841–4.389]	0.106
Hypertension	0.226 [0.029–1.764]	0.156	0.515 [0.026–1.019]	0.663
LVEF	1.127 [0.964–1.316]	0.133	1.126 [0.855–1.482]	0.397
NYHA 3	0.615 [0.180–2.102]	0.439	0.458 [0.008–2.618]	0.705
Obesity	2.283 [0.002–3.248]	0.521	0.759 [0.001–1.621]	0.993
QRS duration	0.951 [0.885–1.021]	0.164	0.897 [0.782–1.016]	0.087
SonR	0.056 [0.001–8.699]	0.263	0.010 [0.001–5.267]	0.520
6MWT	0.992 [0.968–1.016]	0.509	0.973 [0.919–1.031]	0.355
D. Multivariate Cox regression analysis for parameters associated with all cause deaths				
Age	1.782 [1.053–2.302]	0.001	1.362 [0.809–1.780]	0.563
Automatic	1.471 [0.570–3.795]	0.425	1.744 [0.270–2.713]	0.179
Beta blockers	2.009 [0.582–6.490]	0.270	3.338 [0.684–6.781]	0.096
BNP	0.999 [0.996–1.002]	0.434	0.998 [0.994–1.021]	0.258
COPD	0.268 [0.106–0.680]	0.006	2.802 [0.493–5.192]	0.245
CRP	0.918 [0.837–1.007]	0.070	0.915 [0.792–1.057]	0.915
HbA1c	0.832 [0.503–1.742]	0.430	0.587 [0.201–3.105]	0.224
Hypertension	0.027 [0.001–1.801]	0.092	0.898 [0.648–1.547]	0.936
LVEF	1.008 [0.916–1.110]	0.869	0.879 [0.001–5.644]	0.713
NYHA 3	0.292 [0.096–0.888]	0.030	1.966 [0.030–3.082]	0.465
Obesity	2.289 [0.014–3.766]	0.407	0.468 [0.009–1.821]	0.099
QRS duration	0.958 [0.907–1.012]	0.128	0.958 [0.885–1.037]	0.284
SonR	0.191 [0.005–7.540]	0.378	1.684 [0.101–5.647]	0.713
6MWT	1.009 [0.991–1.028]	0.339	1.004 [0.977–1.031]	0.791

Italic values indicate a significant p value (p < 0.05)

^*^ Is for statistical significant (p < 0.05). BNP, B type natriuretic peptide; COPD, chronic obstructive pulmonary disease; CRP, C reactive protein; HbA1c, glycated hemoglobin 1Ac type; LVEF, left ventricle ejection fraction; NYHA 3, New York Heart Association 3 class; SonR, values of SonR signals; 6MWT, 6 min walking test. Multivariate Cox regression analysis for parameters associated with CRT responders

**Fig. 3:**
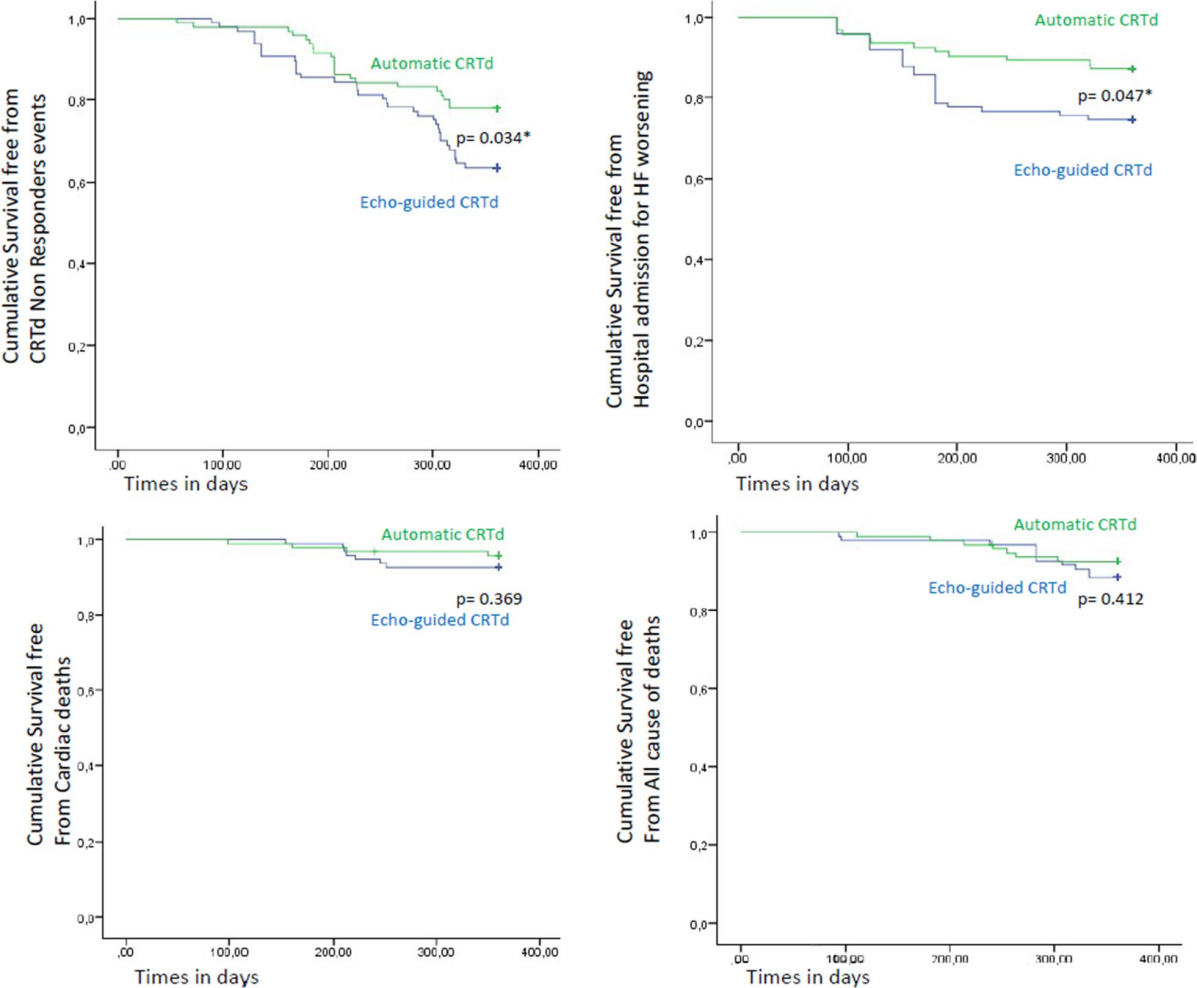
Kaplan curves for “cumulative survival free” at 360 days of follow-up from Cardiac Resynchronization therapy with a defibrillator (CRTd) response (upper left, p < 0.05), hospital admission for heart failure (HF) worsening (upper right, p < 0.05), cardiac deaths (lower left, p > 0.05) and all cause of deaths (lower right, p > 0.05) comparing patients in Automatic CRTd group (green color) vs. Echo CRTd group (blue color). * is for statistical significant (p < 0.05)

Finally, the Kaplan curves showed the cumulative survival free from CRTd non responders, from hospitalization for HF worsening, from cardiac deaths and all cause of deaths in automatic vs. echo group of CRTd patients Fig. 3.

## Discussion

In the present study, we investigated the effects of automatic vs. echo-guided CRTd optimization in patients with T2DM. Thus, in patients with T2DM we reported the ameliorative effects of automatic vs. echo-guided CRTd optimization approach in terms of significant increase of CRTd responders, and of significant reduction of hospital admissions for HF worsening at follow-up end of 12 months (p < 0.05). Notably, for first time in literature we investigated at baseline (CRTd implant) and for all follow-up the values of SonR in the Automatic group of T2DM patients with CRTd. Thus, there were significant modifications of SonR values in CRTd responders vs. non responders patients, and for hospital admissions for HF worsening and for events of cardiac deaths. Finally, and clinically relevant, for T2DM patients the choice of SonR guided automatic CRTd implant could predict a 3.6 folds higher possibility to be CRTd responder. In addition, the patients with higher values of SonR at baseline could have a 2.8 folds higher possibility to become CRTd responders.

Indeed, the automatic lead sensor of right atrium could assess the peak of highest values of atrium pressure [8]. The peak of atrium pressure, as indicated by SonR values, is linked to LV dP/dt max at baseline, and to the endocardial acceleration during LV isovolumetric contraction [8, 10]. Therefore, the amplitude of SonR values is correlated with the heart hemodynamic function, and specifically with the cardiac contractile function [8, 10]. Therefore, we could speculate that modifications of cardiac contractility correspond to modifications of dP/dT values, and to modifications of SonR signals. Furthermore, in HF patients with T2DM the automatic vs. echo-guided CRTd optimization could significantly reduce the levels of inflammatory biomarkers (CRP, IL6, TNFa), and of BNP values via its favorable hemodynamic and clinical effects. The reduction of inflammatory burden, and of BNP values at 6th and 12th month of follow up has been observed in a previous study conducted on T2DM patients with HF and treated by multipolar CRTd [1, 6]. Indeed, both inflammatory markers and BNP are over-expressed in a condition of HF, and in HF patients with depressed cardiac pump [1, 6]. In this context, BNP is a valuable marker of HF, and a predictor of hospitalizations for HF worsening and of worse prognosis in CRTd patients [1, 6, 13]. However, BNP could be relapsed in condition of stable and unstable HF, and used for risk stratification in patients with acute and chronic HF [14]. Therefore, BNP is an independent marker of worse prognosis for patients with the failure of cardiac pump [14], and in those treated with CRTd [1, 6, 13, 15, 16]. Consequently, T2DM patients with severe reduction of cardiac pump, as evidenced by lowest values of LVEF at echocardiography, could experience a worse clinical prognosis [1, 6, 15, 16]. To date, the cardiac pump reduction, in HF patients with T2DM treated with CRTd, could be caused by advanced anatomical degree of ventricular remodeling [15–20], and reflected by the loss of heart synchronism during diastolic and systolic cardiac phases [8, 10]. In this setting, the alterations of AV/IV intervals are linked to, and could mark CRTd patients that evidenced the loss of cardiac synchronism [20]. Indeed, the CRTd patients with highest AV/IV delays could experience worse prognosis by the loss of AV and IV synchronism, and by the worsening of cardiac pump [20]. Therefore, the increase of cardiac pump could lead to the amelioration of clinical outcomes in T2DM patients with CRTd, such as previously observed in overall population of CRTd patients [10]. In addition, we reported an increase of LVEF, with reverse remodeling, and amelioration of NYHA class and clinical status in T2DM patients, which evidenced at baseline highest SonR values, that could be seen as index of best AV/IV synchronism. Thus, we could report that a best optimization of AV/IV delays could lead to best clinical outcomes for CRTd patients [8, 10]. Moreover, in our study we observed and confirmed these results in a selective population of T2DM patients with CRTd. In addition, for first time in literature, we monitored the modification of SonR values for 12 months of follow-up in diabetics with CRTd regards CRTd responders rate, hospitalizations for HF worsening, cardiac deaths and all cause of deaths. Thus, we might speculate that, the automatic vs. echo-guided approach for optimization of CRTd, could be superior to achieve CRTd responders target, and to reduce hospitalizations for HF worsening in patients with T2DM. However, we could summarize the most important functions of automatic CRTd as monitor and activator of cardiac remodeling processes, that are involved in clinical prognosis of CRTd patients. Therefore, it could be relevant to identify at baseline T2DM patients with highest values of dP/dT signals. Indeed, these patients could have lowest AV/IV delays and best cardiac synchronism [18]. Furthermore, it looks intuitive to say that these patients could have a higher possibility to become CRTd responders, and to experience a best clinical prognosis. This point is relevant, because it opens a new scenario in the possibility to identify and to treat at best we can CRTd patients with T2DM at different stages of cardiac dyssynchrony. However, we might speculate to choice specific treatments, that in addition to automatic optimization of AV/IV delays could result in best clinical response in CRTd patients with T2DM. Finally, this could be used to ameliorate CRTd responders and to reduce worse prognosis in failing heart patients with T2DM.

### Study limitations

This study has few limitations. As first, the small sample size and the duration of follow-up could influence study results, that have to be applied in a future study with larger size of T2DM patients, and at more long term follow up analysis. In addition, in the present study by the loss of an experimental animal model of HF with automatic vs. echo guided CRTd, we did not practice cardiac biopsy to show the different inflammation/fibrosis for the main study outcomes. In addition, we did not use a continuous monitoring systems for arrhythmias detection and devices interventions as described by authors [21], and this may affect the study outcomes. Therefore, further studies are needed to better understand the pleiotropic functions of automatic CRTd guided by SonR, and its cardiovascular effects in terms of AV/IV synchronism and best clinical outcomes. Therefore, a larger clinical trial may be adequate to assess all these pathogenic processes in a population of failing heart patients with T2DM treated by automatic CRTd. This may be applied in clinical practice to reduce hospitalizations, and to improve CRTd response in failing heart patients with T2DM.

## Conclusions

Our study results evidenced that automatic vs. echo guided CRTd optimization increased significantly the CRTd responders rate, and reduced hospitalizations for HF worsening in T2DM patients. To date, SonR signals showed a significant modification regards CRTd responders, hospitalizations for HF worsening events, and cardiac deaths. Notably and clinically relevant, the automatic optimization of AV/IV delays could increase of more than 3 folds the possibility to become CRTd responders, and baseline highest values of SonR signals could characterize patients with 2.8 folds higher possibility to become CRTd responders. Therefore, we could suggest to opt for SonaR guided CRTd implant, to reach the best cardiac synchronism, and to increase the possibility for a T2DM patient to become CRTd responder and to reach best clinical prognosis.

## Supplementary information

**Additional file 1: Table S1.** Clinical characteristics of study population at 6th month of follow-up in overall, and automatic vs. echo-guided patients. BMI: body mass index; BNP: B type natriuretic peptide; CRP: C reactive protein; HbA1c: glycated hemoglobin 1Ac type; IL-6: interleukine 6; LVEDd: left ventricle end diastolic diameter; LVEDv: left ventricle end diastolic volume; LVEF: left ventricle ejection fraction; LVESd: left ventricle end systolic diameter; LVESv: left ventricle end systolic volume; NYHA II, III: New York Heart Association II and III class; SonR: values of SonR signals; TNFα: tumor necrosis factor alpha; 6MWT: 6 minutes walking test.** is for statistical significant (p <0.05).

## Data Availability

Data and study materials are available.

## Abbreviations

AV: Atrioventricular
BNP: B type natriuretic peptide
CRP: C reactive protein
CRTd: Cardiac resynchronization therapy with defibrillator
HbA1c: Glycated hemoglobin 1Ac type
HF: Heart failure
ICD: Implantable cardioverter defibrillator
IEGM: Intracardiac electrogram
IL6: Interleukin-6
IV: Interventricular
LV: Left-ventricular
LVEDD: Left-ventricular end-diastolic diameter
LVEDV: Left-ventricular end-diastolic volume
LVEF: Left ventricle ejection fraction
LVESD: Left-ventricular end-systolic diameter
LVESV: Left-ventricular end-systolic volume
NYHA: New York Heart Association
TNF α: Tumor necrosis factor-α
T2DM: Type 2 diabetes mellitus
VTI: Velocity–time integral
6MWT: Six minutes walking test
