# Supplementary material for: Cardiac resynchronization therapy and its effects in patients with type 2 DIAbetes mellitus OPTimized in automatic vs. echo guided approach. Data from the DIA-OPTA investigators
Source: Cardiovasc Diabetol. 2020 Nov 28;19:202. doi: 10.1186/s12933-020-01180-8 (PMC7700711; doi:10.1186/s12933-020-01180-8)
Supplement: Supplementary file 1 — Additional file 1: Table S1. Clinical characteristics of study population at 6th month of follow-up in overall, and automatic vs. echo-guided patients. BMI: body mass index; BNP: B type natriuretic peptide; CRP: C reactive protein; HbA1c: glycated hemoglobin 1Ac type; IL-6: interleukine 6; LVEDd: left ventricle end diastolic diameter; LVEDv: left ventricle end diastolic volume; LVEF: left ventricle ejection fraction; LVESd: left ventricle end systolic diameter; LVESv: left ventricle end systolic volume; NYHA II, III: New York Heart Association II and III class; SonR: values of SonR signals; TNFα: tumor necrosis factor alpha; 6MWT: 6 minutes walking test.** is for statistical significant (p <0.05). [file 12933_2020_1180_MOESM1_ESM.docx]

**Cardiac Resynchronization Therapy and its effects in failing heart patients with type 2 DIAbetes mellitus OPTimized in automatic vs. echo guided Approach. Data from the DIA-OPTA investigators**

Celestino Sardu, MD, MSc, PhD^1^*; Pasquale Paolisso, MD^2^; Valentino Ducceschi, MD, PhD^3^; Matteo Santamaria, MD, PhD4; Cosimo Sacra, MD^4^; Massimo Massetti, MD, PhD^4,5^; Antonio Ruocco, MD, PhD^6^; Raffaele Marfella, MD, PhD^1^

1. Department of Advanced Medical and Surgical Sciences, University of Campania “Luigi Vanvitelli”, Naples, Italy; 2. Unit of Cardiology, Department of Experimental, Diagnostic and Specialty Medicine-DIMES, University of Bologna, Italy; 3. Unit of Cardiovascular Diseases and Arrhythmias, “Vecchio Pellegrini” Hospital, Naples, Italy; 4. Unit of Cardiovascular Diseases and Arrhythmias, “Gemelli Molise”, Campobasso, Italy; 5. Department of Cardiac Surgery and Cardiovascular Diseases, “Catholic University of Sacred Heart”, Rome, Italy; 6. Unit of Cardiovascular Diseases and Arrhythmias, “Antonio Cardarelli” Hospital, Naples, Italy.

**Keywords:** type 2 diabetes mellitus, cardiac resynchronization therapy, automatic CRTd optimization.

**Clinical trial:** ClinicalTrials.gov Identifier NCT04547244

*** Correspondence:**

Prof. Celestino Sardu, MD, MSc, PhD

Department of Advanced Medical and Surgical Sciences,

University of Campania “Luigi Vanvitelli”

Address: Piazza Miraglia 2, 80131, Naples, Italy.

email**:** drsarducele@gmail.com

Tel: +390815665110. Fax: +390815095303

**METHODS**

**CRT-d implant .** The CRTd implant was performed by full trained electrophysiologists (Ce. S., A.R, M. S., V. D), experienced in CRT implantation. All CRT-d implant procedures were standardized to place the right atrial catheters in right atrial appendage, and right ventricular catheters in right ventricle apex, as indicated by anteroposterior, right anterior, and left anterior oblique views projections at radioscopic imaging (1). For all patients we used a multipolar left ventricle (LV) pacing lead, to reach the target left epicardium vessel, and to have the final LV lead position and pacing configuration, with an acceptable pacing thresholds, absence of diaphragmatic stimulation, and chosen anatomic position in the target vessel (1). The final position of the LV pacing lead was assessed by anteroposterior, right anterior, and left anterior oblique views projections at radioscopic imaging (1).

In the **Automatic group** the patients were implanted with a multipolar LV lead, over-the-wire, steroid eluting and with an active fixation bipolar atrial pacing lead (SonRtip, LivaNova, Sorin CRM SAS, Clamart, France), than connected to a quadripolar CRT-d device (Platinium 4LV, 1844 model, Sorin CRM SAS, Clamart, France). In the Echo group the patients were implanted with quadripolar LV pacing leads (Quartet® model 1458Q and Promote Q®, St Jude Medical, Sylmar, CA, USA; Attain Performa® model, Medtronic, Minneapolis, MN, USA), over-the-wire, steroid eluting with a in-line connector connected to an appropriate quadripolar CRT-d device (Quadra Assura CRT-d device, St Jude Medical, Sylmar, CA, USA; Viva® QuadXT and Viva® Quad S cardiac CRT-d, Medtronic, Minneapolis, MN,USA). After CRTd implant and at study follow-up we monitored the functionality of the CRTd system, arrhythmic events, and device interventions (1).

**Control of CRTd pacing in automatic and echo group**

At follow-up, the investigators (Ce. S., A.R, M. S., V. D) performed the CRTd interrogation in automatic and echo group. To date, we applied same protocol of CRTd interrogation for patients in automatic and in echo group. However, for all patients with CRTd we measured the sensing, impedance and pacing thresholds, which are parameters of functionality of the implanted catheters (1). The sensing thresholds were the values of P, and R-wave amplitude; the impedance thresholds were the values of leads’ impedance; the pacing thresholds were the values of lead pacing outputs (1). Thus, we measured and reported the modifications of these 3 parameters at 10 days after clinical discharge, and at 6th and 12th month after CRTd implantation (1). Moreover, the sensing thresholds values were measured from the intracardiac electrograms records, using a sensing configuration (1). The pacing and impedance thresholds values were measured by pacing catheter configurations (1). In addition, in all patients we assessed the percentage of biventricular pacing, arrhythmic events and devices’ interventions (2). Intriguingly, in the automatic group the CRTd system has an automatic optimization program (3). This optimization program uses the signal amplitude variations in different AV/VV delays to calculate the optimized timings automatically, by updating AV/VV intervals weekly (3). Previous study showed a concordance between this automatic and the echocardiographic based method (3).

**Clinical visits, data collection and analysis**

Cardiac deaths, all cause of deaths, hospitalizations for HF worsening were evaluated during clinical visits 10 days after clinical discharge, and after 6th and 12th month by the treating physician, by telephonic interviews, hospital admissions, and discharge schedules (2). Thus, at follow up visits, physicians (PP, AR, VD, Ce. S, M. S) evaluated the New York Heart Association (NYHA) class, and the patients graded their overall condition as unchanged or slightly, moderately, or markedly worsened, or improved since randomization by global self-assessment (4). Indeed, investigators instructed patients how assess regularly body weight, occurrence of dyspnea, and any clinical symptom. In addition, at each visit patients were asked whether medical events or symptoms suggestive of cardiac arrhythmias occurred, and an ECG, an ECG Holter monitoring and the device interrogations were both performed to detect the presence of asymptomatic arrhythmias (1-4). During the clinical evaluations investigators performed physical examination with collection of vital signs, and review of adverse events. A fasting blood (at least 12 h from last meal) was performed for biochemical peripheral blood assay evaluation at every visit.

**Anthropometrics determination**

For the enrolled CRT patients at baseline and during follow up, we evaluated physical examination, vital signs, and review of adverse events. However, we evaluated for each patient the body mass index (BMI) as the ratio between weight in kg and the height squared, as previously reported (6).

**Clinical and Laboratory Parameters**

For each enrolled patients at baseline and for all follow-up duration, laboratory assessment consisted of a complete blood count, blood chemical analysis, coagulation testing, evaluation of liver and renal function, and measures of electrolytes, C-reactive protein. However, authors collected venous blood in EDTA-coated tubes during hospitalization before CRTd implant, and during follow-up.

**Sample size calculation and data collection**

For this study we calculated a sample size with 100 participants for each group, with estimated 80% power to detect a change of 0.015 between the mean MPI of the automatic CRTd-treated and echo-guided CRTd treated groups, at a 5% level of significance. A 20% Loss due to early withdrawals and/or non-evaluable measurements was assumed and, combined with the effect of stratification on analysis, resulted in the requirement to recruit at last 90 patients per treatment group.

The data were collected prospectively from electronic medical records, that were used in clinical setting at participants’ Institutions. For this study authors used electronic systems for data capture, collection, and monitoring, with on-site and real timing data entry. However, after the collection of the patients’ files, all data were analyzed in each participating Institution.

**RESULTS**

In the present study we analyzed 191 T2DM patients with multipolar CRTd, divided in Automatic group (n 93), vs. Echo group (conventional CRTd implant, n 98). At 6th month of follow up, patients in automatic vs. echo group showed a significant reduction of NYHA class, BNP values (156.38 ± 19.26 vs. 203.55 ± 17.26 pg/ml, p 0.018), and inflammatory markers values, with amelioration of 6MWT (314.62 ± 26.73 vs. 238.84 ± 28.12). **Supplementary table**. These effects, comparing automatic vs. echo group of patients, were linked to significant reduction of left ventricle systolic diameters/volumes and mitral valve insufficiency (p<0.05), and to significant improvement of LVEF (35 ± 6 vs. 27 ± 5, p0.001). **Supplementary table**.

**As primary study endpoints,** comparing patients in automatic vs. echo group, we had at 6 months of follow-up a significant higher rate of CRTd responders (68 (73.1%) vs. 58 (59.2%), p value 0.038). **Supplementary table**.

**Supplementary Table.**

| **6 months follow up** |  |  |  |
| --- | --- | --- | --- |
| **PARAMETERS** | **AUTOMATIC**  **(n 93)** | **ECHO GUIDED**  **(n 98)** | **P value** |
| BMI > 30 kg/m^2^(%) | 8 (8.6) | 7 (7.1) | 0.791 |
| Plasma glucose (mg/dl) | 179.8 ± 22.8 | 176.9 ± 21.3 | 0.261 |
| HbA1c (mmol/mol) | 54.9 ± 12.7 | 54.1 ± 11.9 | 0.167 |
| I NYHA class | 5 (5.4) | 2 (2.0) | 0.026* |
| II NYHA class | 41 (44) | 25 (25.5) | 0.013* |
| III NYHA class | 42 (45.1) | 62 (63.3) | 0.006* |
| IV NYHA class | 5 (5.4) | 9 (9.2) | 0.408 |
| QRS duration | 122.1 ±9.5 | 123.2 ± 9.3 | 0.516 |
| 6MWT | 314.62 ± 26.73 | 238.84 ± 28.12 | 0.023* |
| SonR values (g) | 0.93 ± 0.08 | / | / |
|  |  |  |  |
| **Echocardiographic parameters** |  |  |  |
| LVEF (%) | 35 ± 6 | 27 ± 5 | 0.001* |
| LVEDd (mm) | 64 ± 6 | 65 ± 8 | 0.054 |
| LVESd (mm) | 38 ± 4 | 42 ± 5 | 0.001* |
| LVEDv (ml) | 171 ± 25 | 182 ± 31 | 0.051 |
| LVESv (ml) | 111 ± 11 | 132 ± 16 | 0.001* |
| Mitral insufficiency  + (%)  ++ (%)  +++ (%) | 47 (50.5)  41 (44.1)  5 (5.4) | 35 (35.7)  54 (55.1)  9 (9.2) | 0.021*  0.241  0.408 |
|  |  |  |  |
| **Biomarkers** |  |  |  |
| Lymphocytes | 7.29± 2.12 | 8.53 ± 2.17 | 0.001* |
| Neutrophiles | 5.06± 1.82 | 5.41± 1.82 | 0.001* |
| BNP (pg/ml) | 156.38 ± 19.26 | 203.55± 17.26 | 0.018* |
| CRP (mg/L) | 7.29 ± 0.59 | 8.78 ± 0.81 | 0.049* |
| IL6 (pg/ml) | 5.61± 0.03 | 6.19 ± 0.04 | 0.014* |
| TNFα (pg/ml) | 5.36 ± 0.02 | 6.32± 0.02 | 0.007* |
|  |  |  |  |
| **Study outcomes** |  |  |  |
| CRTd responders (%) | 68 (73.1) | 58 (59.2) | 0.038* |
| Hospital admission for HF worsening (%) | 11 (15.1) | 17 (25.8) | 0.084 |
| Cardiac deaths (%) | 1 (1.1) | 2 (2) | 0.061 |
| All cause of deaths (%) | 2 (2.1) | 3 (3.1) | 0.525 |

**Table S1.** Clinical characteristics of study population at 6^th^ month of follow-up in overall, and automatic vs. echo-guided patients. BMI: body mass index; BNP: B type natriuretic peptide; CRP: C reactive protein; HbA1c: glycated hemoglobin 1Ac type; IL-6: interleukine 6; LVEDd: left ventricle end diastolic diameter; LVEDv: left ventricle end diastolic volume; LVEF: left ventricle ejection fraction; LVESd: left ventricle end systolic diameter; LVESv: left ventricle end systolic volume; NYHA II, III: New York Heart Association II and III class; SonR: values of SonR signals; TNFα: tumor necrosis factor alpha; 6MWT: 6 minutes walking test.** is for statistical significant (p <0.05).

**References**

1. Sardu C, Santamaria M, Funaro S, et al. Cardiac electrophysiological alterations and clinical response in cardiac resynchronization therapy with a defibrillator treated patients affected by metabolic syndrome. Medicine (Baltimore). 2017 Apr;96(14):e6558.
2. Sardu C, Paolisso P, Sacra C, et al. Cardiac resynchronization therapy with a defibrillator (CRTd) in failing heart patients with type 2 diabetes mellitus and treated by glucagon-like peptide 1 receptor agonists (GLP-1 RA) therapy vs. conventional hypoglycemic drugs: arrhythmic burden, hospitalizations for heart failure, and CRTd responders rate. Cardiovasc Diabetol 2018;17(1):137.
3. Ritter P, Delnoy PPH, Padeletti L, et al. A randomized pilot study of optimization of cardiac resynchronization therapy in sinus rhythm patients using a peak endocardial acceleration sensor vs. standard methods. Europace. 2012; 14: 1324–1333.
4. Yancy CW, Jessup M, Bozkurt B, et al. 2017 ACC/AHA/HFSA Focused Update of the 2013 ACCF/AHA Guideline for the Management of Heart Failure: A Report of the American College of Cardiology/American Heart Association Task Force on Clinical Practice Guidelines and the Heart Failure Society of America. J Am Coll Cardiol 2017;70(6):776-803.
